# Graph theoretical analysis reveals the functional role of the left ventral occipito-temporal cortex in speech processing

**DOI:** 10.1038/s41598-022-24056-1

**Published:** 2022-11-21

**Authors:** Shuai Wang, Samuel Planton, Valérie Chanoine, Julien Sein, Jean-Luc Anton, Bruno Nazarian, Anne-Sophie Dubarry, Christophe Pallier, Chotiga Pattamadilok

**Affiliations:** 1 Aix Marseille Univ, CNRS, LPL, Aix-en-Provence, France; 2 Aix Marseille Univ, Institute of Language, Communication and the Brain, Aix-en-Provence, France; 3 Cognitive Neuroimaging Unit, INSERM, CEA, CNRS, Université Paris-Saclay, NeuroSpin Center, Gif/Yvette, France; 4 Aix Marseille Univ, CNRS, Centre IRM-INT@CERIMED, Institut de Neurosciences de la Timone, UMR 7289 Marseille, France; 5 Aix Marseille Univ, CNRS, LNC, Marseille, France

**Keywords:** Reading, Language

## Abstract

The left ventral occipito-temporal cortex (left-vOT) plays a key role in reading. Interestingly, the area also responds to speech input, suggesting that it may have other functions beyond written word recognition. Here, we adopt graph theoretical analysis to investigate the left-vOT’s functional role in the whole-brain network while participants process spoken sentences in different contexts. Overall, different connectivity measures indicate that the left-vOT acts as an interface enabling the communication between distributed brain regions and sub-networks. During simple speech perception, the left-vOT is systematically part of the visual network and contributes to the communication between neighboring areas, remote areas, and sub-networks, by acting as a local bridge, a global bridge, and a connector, respectively. However, when speech comprehension is explicitly required, the specific functional role of the area and the sub-network to which the left-vOT belongs change and vary with the quality of speech signal and task difficulty. These connectivity patterns provide insightful information on the contribution of the left-vOT in various contexts of language processing beyond its role in reading. They advance our general understanding of the neural mechanisms underlying the flexibility of the language network that adjusts itself according to the processing context.

## Introduction

Reading acquisition induces massive changes in brain functions, structures, and organization, especially within the auditory and visual systems^1^. The most significant change is the emergence of a new functional role in an area located in the left ventral occipito-temporal cortex (vOT). This area is also labelled as the “Visual Word Form Area” (VWFA) due to its central role in reading^2^. A number of studies have reported that once reading is acquired, the area consistently responds to known scripts, regardless of the characteristic of the writing system^3–5^ and that the degree of activation is dependent upon individuals’ reading ability^6,7^.

Despite its key role in reading, there is empirical evidence that speech processing also involves the left-vOT. Activation in response to speech input was found in various tasks, ranging from those that explicitly require a retrieval of spelling knowledge, such as determining whether spoken words share the same rime spelling^8,9^ or contain a target letter^10,11^, to purely auditory tasks mimicking natural speech processing situations such as spoken word recognition or spoken sentence comprehension^6,12^. These observations raise a question about the general functional role of this area in the language network beyond its well-established contribution to reading.

The contribution of the left-vOT to spoken language processing could be explained by existing theoretical frameworks. According to the *orthographic tuning hypothesis*^1,2^, the left-vOT neurons are progressively tuned to written language input and become specialized in orthographic coding during reading acquisition. Recently, it has also been argued that one factor that contributes to the emergence of this functional selectivity is the pre-existing connectivity between this area and distance areas involved in spoken language processing^13–16^. Thanks to the connection with the spoken language network, despite its selective response to orthographic input in literate populations, the left-vOT could also be activated by spoken input in a top-down fashion, once the spoken input has been converted into its corresponding orthographic code^1,6^. Another framework that explains the functional role of the left-vOT is the *Interactive Account*^17,18^. As argued by Price and Devlin^17,18^, the neural population within the left-vOT would respond to both orthographic and to non-orthographic inputs such as spoken words, objects, colors, or braille script. According to the authors, the area is not specific to orthographic processing but supports multiple functions depending on its interaction with other regions: Without being functionally selective, the prominent function of this area in reading would arise from its ideal location at the transition between the occipital and the temporal lobe, enabling unique interactions between visual and language regions.

Thus, although the two theoretical frameworks disagree on the degree of functional selectivity of the left-vOT to orthographic input, both assume that the left-vOT acts as an interface between the visual system and areas involved in language processing^1,18^. This view is coherent with empirical evidence that the left-vOT is connected with widely distributed regions through intrinsic connectivity^19–23^ and anatomical connections^22,24,25^. Anatomical evidence shows that the left-vOT is connected to multiple language regions, including the perisylvian language areas^24^, the inferior frontal gyrus, inferior parietal cortex, and anterior temporal lobe^25^. Studies using functional connectivity confirm the preferential connections between the left-vOT and language regions^19,21–23^, while more distributed prefrontal, parietal, and bilateral connections are also reported^19,20,22,26^.

So far, studies on the functional role of the left-vOT have mainly focused on reading tasks. Studies that used spoken materials only reported modulations of BOLD signal in the area in different speech processing conditions (using univariate activation) without examining its role from the functional connectivity perspective. To fill this gap, here we proposed to investigate the functional role of the left-vOT during spoken language processing by modeling the brain as a network^27,28^. This analysis approach should allow us to test the hypothesis that, in addition to its key role in reading, the left-vOT, may also act as an interface that coordinates the communication between different brain regions and sub-systems during spoken language processing and that its functional role may depend on processing context. To this end, we applied graph theoretical analysis to fMRI data from the study of Planton et al.^12^ collected while adult participants performed either perception (P: decide whether the same sentence was presented twice in a row) or comprehension task (C: decide whether a statement is true) on spoken sentences. Within each speech processing task, spoken sentences were presented either against a silent background (N-) or against unintelligible “multi-speaker” babble noise, mimicking a “cocktail party” situation (N +). Planton et al.^12^ reported that these manipulations of task demands and quality of speech signal had significant impacts both on performance and brain activation across different brain areas, including the left-vOT, although the area was independently identified in a visual-word localizer task. Here, we are interested in examining the possible impacts of these manipulations on different measures of graph theoretical analysis described below.

Graph theory provides a quantitative tool to investigate the organization of brain networks and interactions between brain regions^29,30^. In the present study, brain networks were constructed as a graph consisting of nodes and edges, in which nodes are regions of interest (ROIs) and edges are beta-series connectivity between ROIs^31^. The organization of brain networks and the functional role of the left-vOT within the networks were examined in different processing contexts which included a “baseline” where participants were passively exposed to multi-speaker babble noise and four “active” speech processing conditions where participants conducted the two sentence processing tasks in the two types of background as described above.

The graph theoretical analysis was conducted in two steps. First, we described the organization of the brain networks at the global scale (by contrasting the active conditions to the baseline) and compared the global metrics of the brain networks. This initial analysis allowed us to explore whether there is a difference between speech processing conditions before conducting the main analysis at the nodal level. An observation of a significant difference between conditions would indicate that the task demands and/or the quality of speech signal that we manipulated can influence the global organization of the brain networks. In this case, the analyses conducted on the nodal measures should take into account the global measures. On the contrary, the absence of difference between conditions at the global scale would allow us to directly compare the nodal measures of the left-vOT between speech processing conditions. The analyses conducted at the nodal scale focused on the left-vOT. They aimed to test whether the area acted as an interface that coordinates the communication between distributed brain regions and sub-networks during spoken language processing and whether its functional role varied across speech processing conditions. Specifically, the global measures *global efficiency* and *clustering coefficient* respectively characterize the functional integration and segregation of the network. Higher *global efficiency* indicates higher level of information exchange across the whole brain while higher *clustering coefficient* indicates increased information exchange within local clusters of neighboring nodes. Moreover, the modular organization of the network was estimated by *Modularity Q*, which expresses how well a network can be subdivided into non-overlapping sub-networks. After examining the global topology, we specified the functional role of the left-vOT within the network using different nodal measures. Three nodal measures are of particular interest*,* i.e., *flow coefficient*, *betweenness centrality* and *participation coefficient*, that allow us to specify whether the left-vOT acts as an interface in the network, also named bridge or connector in terms of graph theory. *Flow coefficient* estimates the capacity of a node to transfer information between its neighbors. Nodes with large *flow coefficient* are identified as “local bridges” that coordinate the communication between their neighboring nodes in the network^32^. *Betweenness centrality* estimates how often a node joins the shortest path between pairs of nodes in the network^33^. Nodes with large *betweenness centrality* are “global bridges*”* that coordinate the global information exchange between distributed nodes in the network. On the basis of the connections between a node and sub-networks, *participation coefficient* indicates whether the node acts as a “connector” that coordinates the communication between different sub-networks in the network^33,34^ (see Supplementary Table S1 for the interpretations of graph measures).

## Results

### Global network changes induced by speech processing

**Figure 1:**
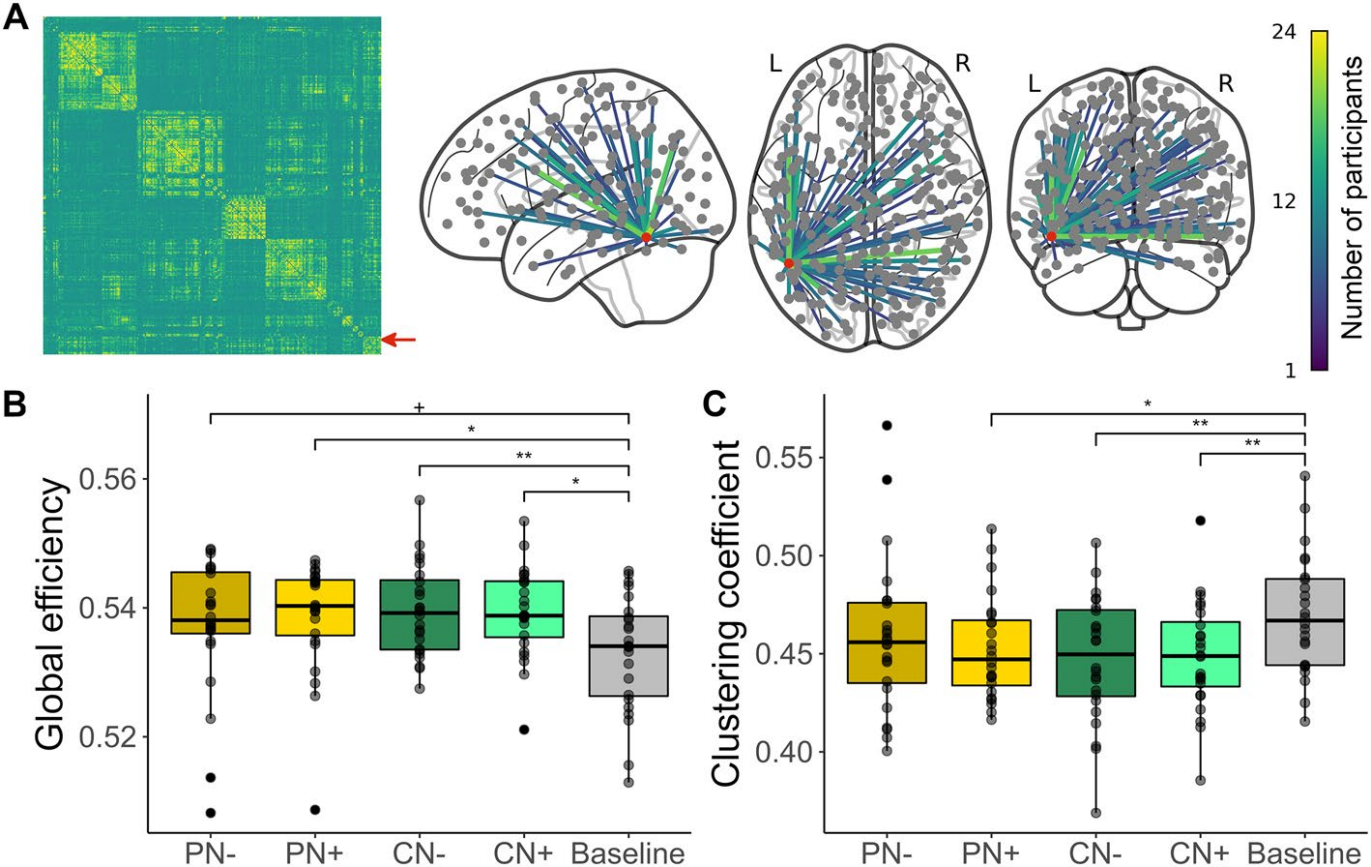
Overall network organization and global topology. (**A**) The network of the baseline was used as reference to illustrate the overall network organization. The baseline network is shown as a matrix of accumulated individual networks (left panel). The connections of the left-vOT node (indicated by the red arrow) are extracted from the accumulated baseline network and shown on the glass brain (right panel, the red dot represents the left-vOT node. The connections present in more than 25% of participants are shown). (**B)** The speech processing conditions showed higher *global efficiency* than the baseline. (**C)** The speech processing conditions showed lower *clustering coefficient* than the baseline. **: p < 0.01; *: p < 0.05; + (marginal): p < 0.06. Boxplot shows the range of the values (vertical line), the interquartile range (box) and the median (horizontal bold line). Each dot corresponds to a participant. PN-: perception clear speech; PN + : perception speech-in-noise; CN-: comprehension clear speech; CN + : comprehension speech-in-noise.

Figure 1A illustrates the overall organization of the brain network (left panel), as well as the pattern of the connections of the left-vOT node in the baseline (right panel). The baseline network showed a modular organization, where the left-vOT node was connected with widely distributed brain regions as shown in the glass brain.

As illustrated in Fig. 1B and 1C, conducting an active task induced significant global network changes in terms of both *global efficiency* and *clustering coefficient*. Compared to the baseline network, the networks of the speech processing conditions showed an increase in *global efficiency* (*p* < 0.0024; Fig. 1B). The *post-hoc* pairwise comparisons showed that the *global efficiency* of the PN + , CN- and CN + conditions are significantly higher than the baseline (all *post-hoc p*s < 0.016), while the difference between the PN- and the baseline is marginally significant (*post-hoc p* < 0.056). These results indicate an overall higher level of communication between brain regions during speech processing in comparison to the baseline. This increase in functional integration was also accompanied by a decrease of functional segregation as revealed by the reduction of *clustering coefficient* (i.e., the reduced interconnections between topological local neighbors) in the speech processing conditions compared to the baseline (*p* < 0.0059; Fig. 1C). *Post-hoc* comparisons showed that the reduction was mainly induced by speech processing in the PN + , CN- and CN + conditions (PN-: *post-hoc p* > 0.1; PN + : *post-hoc p* < 0.025; CN-: *post-hoc p* < 0.0074; CN + : *post-hoc p* < 0.0078). No significant differences were found between the four speech processing conditions in either *global efficiency* (all *post-hoc p*s > 0.30) or *clustering coefficient* (all *post-hoc p*s > 0.15).

In addition, the modular organization of the networks was characterized by *number of communities (sub-networks)* and *modularity Q*. No significant differences were found across the baseline and speech processing conditions in either *modularity Q* (*p* > 0.39) or *number of communities* (*p* > 0.097). The community structures were further compared across the baseline and speech processing conditions by using *normalized mutual information* (NMI) as a similarity measure. The MNI values in all pairwise comparisons were higher than 0.73 (the range of NMI is from 0 to 1). These results suggested that the modular organization is generally consistent across the baseline and speech processing conditions and the reconfigurations of sub-networks only involve a limited number of brain regions.

Overall, the analyses conducted at the global scale suggested that speech processing led to a more integrated and less segregated network while the modular organization remained constant regardless of task demands and quality of speech signal. Having ensured that the networks of the four speech processing conditions did not differ significantly in terms of global topology, in the next step, we addressed the main issue of the present study, which is the functional role of the left-vOT in speech processing.

### Nodal topology of the left-vOT node

**Figure 2:**
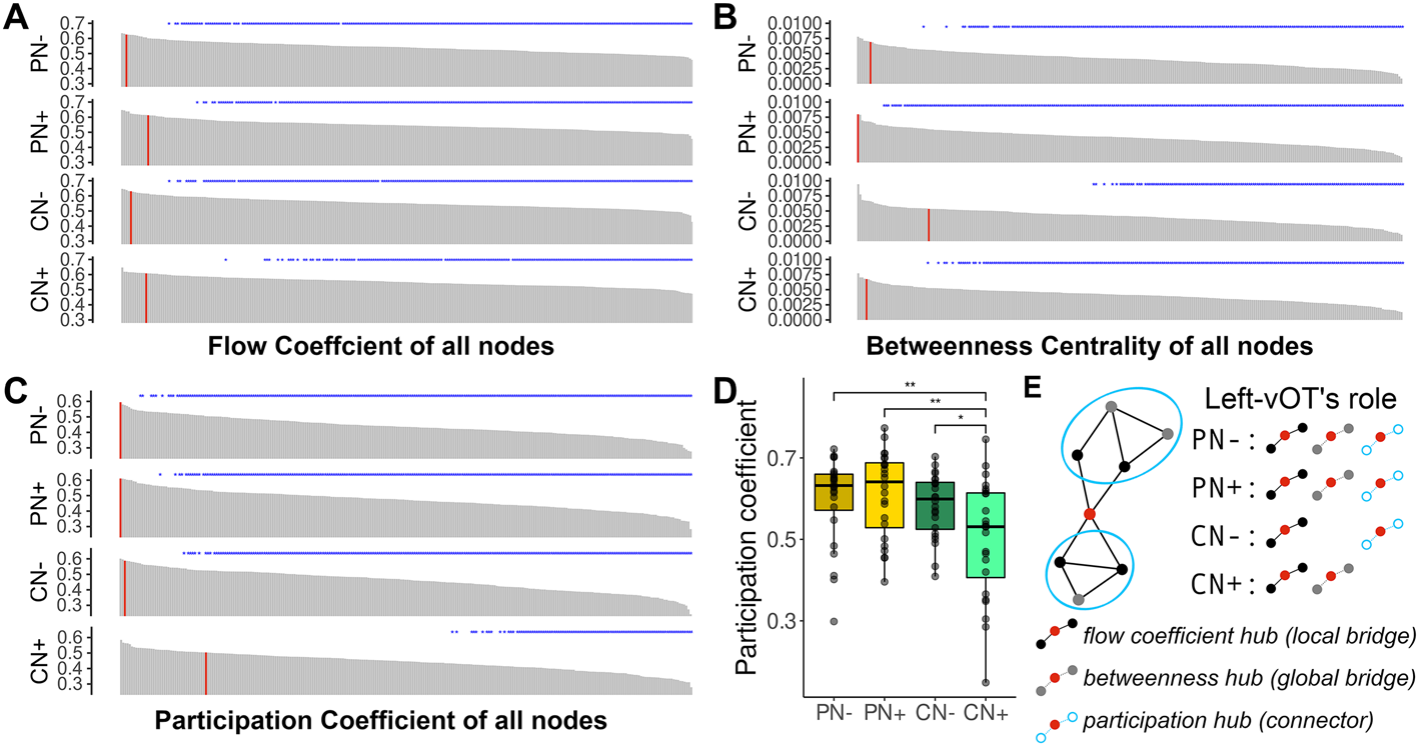
Nodal topology of the left-vOT node. (**A)** Across the four speech processing conditions, the *flow coefficient* of the left-vOT node was ranked 3rd (PN-), 13th (PN +), 5th (CN-), and 12th (CN +) among the 263 nodes. The vertical red bar indicates the rank of the left-vOT node. The blue asterisks indicate the nodes whose values were significantly lower than the left-vOT’s (paired permutation test, *p* < 0.05 unc.). (**B**) The *betweenness centrality* of the left-vOT node was ranked 7th (PN-), 1st (PN +), 35th (CN-), and 5th (CN +) among the 263 nodes. (**C**) The *participation coefficient* of the left-vOT node was ranked 1st (PN-), 1st (PN +), 3rd (CN-), and 40th (CN +) among the 263 nodes. (**D**) The *participation coefficient* of the left-vOT node was significantly lower in the CN + condition than in the PN-, PN + and CN- conditions. (**E**) The diagram illustrating the role of the left-vOT (red dot) as a local bridge that supports the communication between neighboring nodes (black dots), as a global bridge that supports the communication between remote nodes (gray dots) and as a connector that supports the communication between sub-networks (blue circles).

To characterize the functional role of the left-vOT node in the different speech processing conditions, three nodal measures, i.e., *flow coefficient*, *betweenness centrality,* and *participation coefficient*, were estimated to identify a node as local bridge, global bridge, and connector, respectively. For each nodal measure, the values were averaged across all participants and were used to rank the 263 nodes considered in the analysis (see Methods for the selection of nodes). For each nodal measure, nodes ranked in the top 5% (above 13th out of 263) were then identified as hubs. In terms of *flow coefficient*, the left-vOT was identified as a local bridge for all speech processing conditions (above 13th; Fig. 2A), without any differences between the conditions (*p* > 0.63). In terms of *betweenness centrality*, it was a global bridge in PN-, PN + and CN + (above 7th), but not CN- (35th; Fig. 2B), with a marginal significant between-condition difference (*p* < 0.068). Based on the community structures, *participation coefficient* was estimated and adopted to assess whether the left-vOT acted as a connector between sub-networks. In PN-, PN + and CN- conditions, the left-vOT node was identified as a connector with high *participation coefficient* (above 3rd), whereas its rank in the CN + condition was dropped to 40th (Fig. 2C). The between-condition comparisons revealed a significant difference in *participation coefficient* (*p* < 0.0012; Fig. 2D). The *post-hoc* tests confirmed that the *participation coefficient* in the CN + condition was significantly lower than in the PN- (*p* < 0.0059), PN + (*p* < 0.0034), and CN- (*p* < 0.028) conditions.

To summarize, in the PN- and PN + conditions, the functional role of the left-vOT as a bridge and connector was revealed by the three measures (i.e., *flow coefficient*, *betweenness centrality* and *participation coefficient*; Fig. 2E), indicating its consistent role in coordinating the communication between neighboring and distributed regions and sub-networks during speech perception. In the CN- condition, the left-vOT was not identified as a global bridge due to its *betweenness centrality* being reduced, but it still played a role as local bridge and connector. Finally, the left-vOT’s functional role changed again in the CN + condition. The area acted as a local and global bridge but not a connector (Fig. 2E).

As a control, we computed the same nodal measures on a ROI within the primary visual cortex and a ROI in the left posterior STG, the latter being involved in both spoken and written language processing (Supplementary Table S2). Both ROIs showed lower ranks and significantly lower values in different nodal measures compared to the left-vOT (all *p*s < 0.012) except in *participation coefficient* in the CN + condition (*p* > 0.11) where the left-vOT itself did not act as a connector (see Supplementary Fig. S1 and S2). This result suggests that the pattern of connectivity observed in the left-vOT did not generalize either to a primary area in the visual system or to a cross-modal area like pSTG in the auditory system. Finally, the global and nodal results reported here were further validated across a range of densities (see Methods) and using a symmetrical set of ROIs which allowed us to ascertain that the main results are robust across densities and sets of ROIs (see Supplementary Results 1).

Altogether, this finding suggests that both task demands and quality of speech signal induced changes in the left-vOT’s functional role. In the perceptual task, the left-vOT systematically acts as a hub that coordinates the communication at the local, global and sub-network levels regardless of the quality of speech signal. However, its role changes in the comprehension task and also becomes dependent on the quality of speech signal as indicated by a significant difference between the speech processing conditions, especially on the value of *participant coefficient* (Fig. 2D and 2E). The analyses presented below further investigated the impacts of speech processing conditions, firstly by looking at the relationship between *participation coefficient* of the left-vOT and task performance; and secondly, by examining whether the left-vOT belongs to the same sub-network in the different processing contexts.

### Relationship between participation coefficient of the left-vOT and task performance

**Figure 3:**
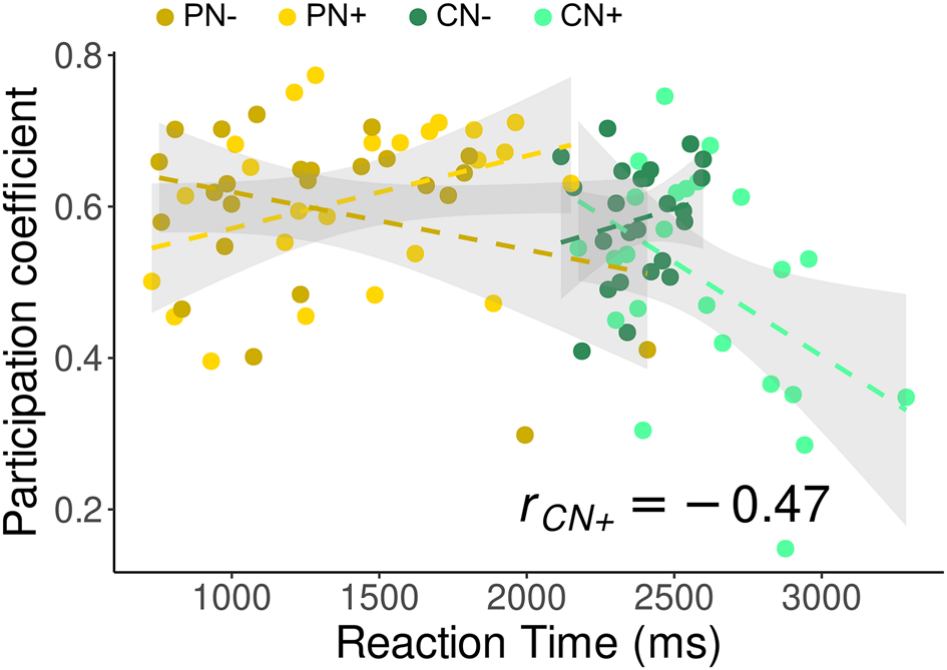
The *participation coefficient* and reaction time were negatively correlated in the CN + condition (light green dots and dashed line; Pearson’s r = − 0.47, *p* < 0.022), but not in the other conditions (all *p*s > 0.078).

As mentioned above, the left-vOT’s *participation coefficient* was lowest in the CN + condition. Interestingly, this observation mirrored the task performance reported in Planton et al.^12^, which was also poorest in the CN + condition, both in terms of accuracy (PN-: 96%, PN + : 92%, CN-: 88%, CN + : 63%) and reaction time (PN-: 1296 ms, PN + : 1413 ms, CN-: 2389 ms, CN + : 2575 ms). This possible relationship between *participation coefficient* and task performance was further examined in a correlation analysis. As shown in Fig. 3, the correlation analysis showed that *participation coefficient* and reaction time was negatively correlated in the CN + condition (Pearson’s r = -0.47, *p* < 0.022), but not in the other conditions (all *p*s > 0.078). This result indicates that participants who responded more quickly in the speech comprehension task conducted in a noisy environment were those who showed higher level of communication between the left-vOT and other sub-networks.

### Identification of the left-vOT’s sub-network in different speech processing contexts

**Figure 4:**
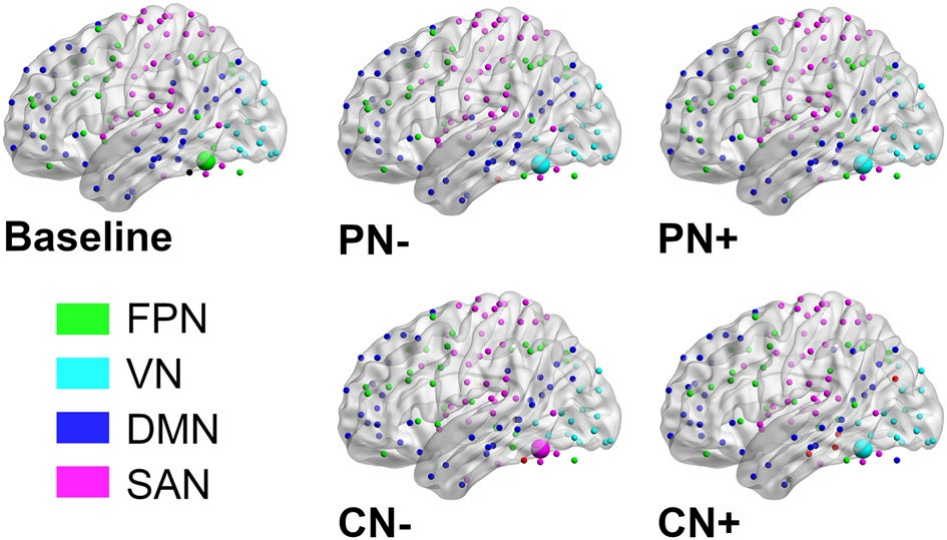
The baseline network and the four speech networks have similar community structures, which consist of visual network (VN, colored cyan), fronto-parietal network (FPN, colored green), default mode network (DMN, colored dark blue), and sensorimotor-auditory network (SAN, colored magenta). The brain maps illustrate that the left-vOT node (the largest bubble in each brain map, whose color refers to the corresponding sub-network) belonged to different sub-networks in different conditions. The brain maps were visualized using the BrainNet Viewer^36^.

In addition to the left-vOT’s *participation coefficient*, for the baseline and each of the four speech processing conditions, the community structures were detected by subdividing the whole brain network into several sub-networks through relatively maximizing intra-connections and minimizing inter-connections. This analysis allowed us to identify the partitions of the sub-networks and the specific sub-network that the left-vOT belonged to. Based on the sub-networks defined by a meta-analysis^35^, four sub-networks were identified for both baseline and speech processing conditions (Fig. 4; see Supplementary Results 2 for details on sub-network labeling): visual network (VN), fronto-parietal network (FPN), default mode network (DMN), and sensorimotor-auditory network (SAN).

As illustrated by the largest bubble in each brain map presented in Fig. 4, the left-vOT participates in different sub-networks in different processing contexts. Specifically, in the baseline, the left-vOT was a part of the fronto-parietal network (FPN). When the participants performed the perceptual task without noise (PN-), the area became part of the visual network (VN), and it remained in this network even when the background noise was added (PN +). Interestingly, the area switched from the visual network to the sensorimotor-auditory network (SAN) in the comprehension task when the participants had to extract the semantic content of clearly presented spoken sentences (CN-). However, the area disengaged from the SAN and returned to the VN when the background noise was added during sentence comprehension (CN +), which made the task more difficult (as illustrated in task performance). It is noteworthy that this pattern of left-vOT sub-network switching was obtained at the sparsest density where the noise in network connections were kept at a minimal level (see Methods).

## Discussion

The present study investigated the functional role of the left-vOT in speech processing by applying graph theoretical analysis to fMRI data collected during spoken sentence processing in different conditions, as defined by task demands and quality of speech signal^12^.

At the global scale, the measures of *global efficiency* and *clustering coefficient* showed that, compared to passive exposure to unintelligible conversation noises, processing intelligible speech led to a more integrated and less segregated network. This evidence of an overall increase of information exchange across the whole brain is coherent with existing observations that processing speech recruits highly distributed brain areas that are involved in the analyses of acoustic, phonological, semantic, and syntactic information^37,38^. The reorganization of the global network toward distributed processing observed here could be accounted for by the fact that, in the present protocol, participants were required to process entire sentences either to decide whether the same sentence was presented twice in a row or to extract their meanings. Similar global network reorganizations have indeed been reported in other studies involving sentence reading and comprehension^39,40^. Importantly, the fact that there was no difference between the four speech processing conditions at the global scale allowed us to directly examine the role of the left-vOT between these conditions at the nodal scale under the context of a similar global network organization.

At the nodal scale, the analyses focusing on the left-vOT provided evidence supporting the hypothesis that, during spoken language processing, this “reading area” consistently supports the communication between different parts of the brain by acting as a bridge and/or a connector. However, its precise functional role varied depending on task demands and quality of speech signal. As summarized in Fig. 2E, the ranks of the left-vOT compared to the other nodes in the network suggest that in the speech perception task, regardless of the presence of background noise, the area contributed to the communication between neighboring brain areas, remote brain areas and sub-networks, by acting as a local bridge (*flow coefficient* hub), a global bridge (*betweenness centrality* hub), and a connector (*participation coefficient* hub), respectively. However, the result pattern became more complex in the comprehension task, which indicated the flexibility of the role of the left-vOT during speech processing. It is also worth noting that this pattern of connectivity observed in the left-vOT did not generalize either to a primary area in the visual system or to a cross-modal area like pSTG in the auditory system.

As was the case in the perception task, during active speech comprehension, the left-vOT preserved its role as a local bridge regardless of the quality of speech input, thus indicating its stable functional role in coordinating communication at least at the local level. This observation suggests that during spoken language processing the area systematically supports information exchange between its neighboring nodes, which otherwise would likely remain isolated from each other^32^. This role of an interface between neighboring regions could be explained by the location of the left-vOT at the transition between the occipital and the temporal cortex, as well as the fact that it lies along several major white fibers^24,25^.

Interestingly, the quality of the speech input presented during the comprehension task plays an important role in determining whether the left-vOT would act as a global bridge between widely distributed brain areas or as a connector between sub-networks. When speech input was clearly audible (CN-), the measure of *participation coefficient* indicated that the left-vOT acted as a connector supporting the communication between different sub-networks and, as will be more extensively discussed below, the area became part of the SAN rather than of the VN as in the other speech processing conditions. Finally, when participants had to extract meanings from degraded speech input (CN +), which also led to the lowest performance in terms of both accuracy and reaction time, the left-vOT no longer acted as a connector between the SAN and the other sub-networks. It turned back to the VN and acted as a global bridge, i.e., it tended to join the shortest path between pairs of nodes in the brain network and contributed to the coordination of the global information exchange between remote areas^33^. However, even though the presence of background noise in this task clearly affected the pattern of connectivity, the finding obtained so far does not allow us to conclude whether this change was induced by the presence of noise per se or by other cognitive operations recruited during difficult speech processing situations (for instance, see the discussion on the possible role of attention below).

In addition to the above description of the functional role of the left vOT in different speech processing conditions, the analysis of the community structures also revealed interesting observations. Overall, the same sub-networks were identified in all processing contexts. They consist of fronto-parietal network (FPN), default mode network (DMN), visual network (VN), and sensorimotor-auditory network (SAN). The stable partition of sub-networks across the different processing contexts might reflect a common meso-scale organization of the brain network^41^ engaged by both intelligible and unintelligible speech processing^42^. The FPN, DMN and VN are canonical sub-networks that have been consistently revealed by previous studies using functional connectivity^35,43–45^. Interestingly, the SAN, which covers auditory and sensorimotor cortex, is more specifically considered as the main sub-network for auditory and speech processing, which is in line with the findings that sensorimotor cortex is also involved in speech perception and comprehension^46–49^.

Despite the consistency of the sub-network partition across conditions, the results showed that the sub-network to which the left-vOT belonged varied depending on task demands and the quality of speech signal. The left-vOT affiliated with the FPN in the baseline where unintelligible speech noises were presented without any task demands. This result is in line with previous studies that showed strong intrinsic connectivity between the left-vOT and fronto-parietal regions^20,22^. Interestingly, using Independent Component Analysis on intrinsic activity extracted from an auditory lexical decision task, López-Barroso et al.^50^ also found that the left-vOT is the only region that belonged to both the left fronto-parietal network and the lateral visual network. These findings, together with our results, suggest that the left-vOT could be a part of the fronto-parietal system in task-free situations such as resting-state or passive exposure to noise, while it might also maintain a subtle link with the visual system^50^.

Here, we showed that this subtle link with the visual system became obvious when participants perceived spoken sentences, regardless of the quality of speech signal. As illustrated in Fig. 4, the left-vOT belonged to the VN, and the high values of participation coefficient (Fig. 2C) showed that it interacted with the other sub-networks at the highest level compared to the other regions. These observations indicate that, when one listens to speech, the left-vOT might act as a connector that links the visual system and the other sub-systems. Interestingly, when speech comprehension was explicitly required, the left-vOT not only adapted its functional role but also changed a sub-network to which it belonged depending on the quality of speech signal. More specifically, when the sentences were clearly audible (no background noise) and speech comprehension could be performed without difficulty, the left-vOT still acted as a connector, but it became part of the SAN and no longer acted as a global bridge that contributes to the communication between remote areas. Thus, as part of the SAN and by disengaging from the global information exchange between distant areas, the left-vOT might be more strongly involved in speech processing and in the coordination of communication between the spoken language system and the other sub-systems. On the contrary, when speech comprehension was compromised due to background noise (CN +), the area abandoned its role as a connector. It returned to the VN and resumed its role as a global bridge (Figs. 2E and 4). The disengagement of the left-vOT from the spoken language system, as reflected by the change of sub-network (from the SAN to VN) and functional role (from connector to global bridge) in the most difficult speech comprehension situation (CN +), mirrors some previous findings that the degree of activation of some higher-order areas in the language network does not increase linearly with task difficulties^51,52^. For instance, Obleser et al.^51^ used a speech perception task in which they manipulated the clarity (S/N ratio) and the semantic predictability of speech signal and found that the benefit of semantic predictability was strongest at an intermediate level of speech degradation. The improvement in comprehension of degraded speech thanks to the semantic predictability was associated with an increase in activity and functional connectivity between higher-order cortical areas. In line with our observation, such benefits and thus, the involvement of higher-order cortical areas disappeared when the signal was severely degraded. However, at the present stage of research, our hypothesis on the disengagement of the left-vOT from the spoken language system when speech processing performance declines beyond a critical point needs to be confirmed by a specific protocol that aims at testing this issue explicitly.

As briefly discussed earlier, task performance in the CN + condition also decreased significantly compared to the other conditions both in terms of accuracy and processing speed, which indicates an increase of task difficulty and cognitive demands for speech comprehension in a noisy environment^53^. Related to this observation, we observed a negative correlation between the reaction times and the *participation coefficient*. The correlation indicates that, although the left-vOT is no longer a connector at the group level, participants who maintained higher level of communication between the left-vOT and other sub-networks also tended to have less difficulties in comprehending the sentences despite the background noise. Taken together the patterns of connectivity in the different speech processing situations, our finding suggests that, when the left-vOT acted as a connector, the participation coefficients that were generally high across individuals did not vary with the task performance. However, when it did not act as a connector, the links between the participation coefficients (whose values became more varying) and the task performance became more obvious in terms of individual differences. This link between task performance and the pattern of left-vOT connectivity is of major interest and deserves further investigation since it could inform us about the role of this “reading area” and the benefit of learning to read on speech processing performance as previously reported in the literature^1,54,55^.

Together, the complex pattern of connectivity of the left-vOT and its potential relationship with task performance reported here is in favor of the idea that, in addition to its central role in reading, this area is also an interface that adaptively coordinates the communications between different brain regions and sub-systems during spoken language processing. This role could be supported by the left-vOT’s intrinsic and anatomical connections with various brain regions and systems^19–25^, and, as shown here, is modulated by the interaction between bottom-up (quality of the signal) and top-down (task demands) information. At the theoretical level, the idea that the left-vOT acts as interface between the visual system that conveys bottom-up sensory inputs and non-visual systems that provide top-down information on non-visual stimulus attributes has been the keystone of the *Interactive Account* of left-vOT function^17,18^. More recently, studies conducted within the framework of the *orthographic tuning hypothesis* also provided evidence along this line^13,14,56^. So far, various observations regarding the structural and functional connectivity of the left-vOT have indeed been reported in the literature. These observations are in line with the assumptions that the function of the left-vOT varies depending on stimulus, experience-dependent learning and processing context, and that reading process itself involves different cortical regions whose degree of contribution depends on the task in hand. Our finding is totally in accordance with this view. It provides additional evidence that such flexible and adaptive functional role of the vOT is not restricted to reading or visual input processing but also applies during spoken language processing.

Finally, although we have discussed the functional role of the left-vOT as part of the language network, it is worth mentioning that the manipulation of task demands and quality of speech signal inevitably affects the level of attention demand that may increase with task difficulty. As also suggested by several studies, the left-vOT could be a part of the attention system^20,57^ and plays a role in integrating language and attention^22^. In this context, one may consider that the changes of the functional role of the area might to some extent reflect the modulation of attention demand in different speech processing situations. However, a recent study showed that both attended and unattended spoken language yielded higher activation in the anterior and middle left-vOT than auditory controls^58^. In the present protocol, the level of attention in the different conditions could not be measured. Thus, even though the potential role of attention deserves further consideration in future studies, one cannot claim that the modulation of the functional role of the left-vOT during different speech processing contexts examined here could be attributed to attention per se.

In conclusion, as a complement to the previous observations of the activation of the left-vOT during speech processing^8–12^, our study is the first to apply graph theoretical analysis to examine its role in terms of functional connectivity by considering it as an “interface area” in speech processing. The results indicate that the left-vOT adapts its role in the network to support the communication between distributed brain regions and sub-systems according to task demands and quality of speech signal. These varying patterns of functional connectivity provide empirical evidence that pave the way to further explore the role of the left-vOT in different processing contexts from a network perspective^27,28^, both within and outside the language domain^2,18^. More studies are nevertheless needed to further explore this initial observation, for instance, whether the adaptive role of the left-vOT is supported by a single population of neurons that adjusts its pattern of connectivity according to the processing context, or by different subpopulations of neurons located in the same area that may have different pattern of connectivity^59^. Also, using measures of neural propagation with higher temporal-resolution and a causal interventional approach^60,61^ could help to clarify the temporal dynamics, the direction of the functional connectivity between the left-vOT and the other brain areas, as well as the causal relationship between the pattern of brain connectivity and language processing performance.

## Methods

### Participants

Twenty-four native French speakers were recruited in the study (mean age: 24.05 ± 3.46, 11 females). Participants were healthy, right-handed, with normal hearing and vision and reported no past or current neurological or language disorders. Written informed consents were obtained from all participants. The study was approved by the local ethics committee (CPP Sud Méditerranée #RCB 2015-A00845-44). All experiments were performed in accordance with relevant guidelines and regulations.

### Tasks and Stimuli

#### Spoken sentence processing tasks

The stimuli were spoken sentences expressing true or false statements. Both tasks used a Go/NoGo paradigm. In the perception task, participants were instructed to press the response button as soon as they heard the same sentence twice in a row (Go trials). In the comprehension task, they had to press the response button whenever they heard a false statement, thus requiring a complex semantic analysis (Go trials). For both tasks, all NoGo trials were true statements. The two tasks were alternately presented in four separate runs (two runs per task). The run order was counterbalanced across participants. Each run lasted 7.2 min. and contained 10 Go trials and 70 NoGo trials that were pseudo-randomly distributed into 20 blocks of 4 trials. Two consecutive go trials were avoided. In half of the blocks, the spoken sentences were presented against clear background and half were presented against unintelligible multi-speaker babble noise at an SNR of + 6 dB. A preliminary experiment was conducted on an independent group of twelve participants using recorded scanning noise to ensure that the multi-speaker babble noise can be distinguished from the scanning background noise and that the volume of the speech signal in the no-noise conditions was high enough to allow the participants to easily recognize the speech sound despite the noise of the scanner. An extensive description of the construction of the material can be found in Planton et al^12^. In each participant, each NoGo sentence was presented only once but across participants, it appeared equally in the four active listening conditions (perception of clear speech, PN-; perception of speech-in-noise, PN + ; comprehension of clear speech, CN-; comprehension of speech-in-noise, CN +). In addition to these “active” blocks, 5 “rest” blocks corresponding to silent background and 5 “rest” blocks corresponding to the multi-speaker babble noise were added to the run. Each of the active and rest blocks lasted 14 s on average (range 12 s–18 s). Within each run, the order of the blocks from the different conditions was pseudorandomized to avoid two consecutive blocks of the same condition. At the trial level, each spoken sentence lasted from 1 s-2.4 s. During this period, a visual fixation cross was presented on the screen. After the sentence presentation, there was a blank screen whose duration was jittered. The SOA (3.55 s on average) followed an exponential curve to maximize design efficiency. The same procedure was adopted during the rest trials, except that the sentence was replaced by silence or multi-speaker babble noise.

#### Visual localizer task

To individually localize the left-vOT, the participants performed a functional localizer task in which sequences of 6-letter mono or disyllabic words and 6-letter consonant strings were visually presented and participants were required to detect, by pressing the response button, twelve target stimuli (Go trials: “######”) that were randomly included in the sequences. The task was presented in a single run that lasted 7.4 min. During the run, words and consonant strings were grouped in short blocks of ~ 12 s each (range 11 s–13.3 s). Each block contained 24 stimuli of the same category. Altogether, there were 12 word-blocks and 12 consonant-string blocks. For both categories, each stimulus remained on the screen for 340 ms and was followed by a blank screen of variable duration (~ 160 ms on average). In addition to these 24 “active” blocks, 12 “fixation” blocks during which a cross remained on the screen throughout the block duration (~ 12 s on average) were also included. The 36 blocks were presented in a pseudorandom order to avoid repetition of the same condition. For all stimulus types, the visual input always appeared in the center of the screen, in white font on a dark grey background. Word stimuli were nouns and adjectives selected from the French database LEXIQUE (http://www.lexique.org) with lexical frequency ~ 7.21 per million on average. No words or consonant strings were presented twice during the run. The analysis conducted in the visual localizer task is presented in the SI, Methods A.

### fMRI Data Acquisition and Pre-processing

The experiment was conducted on a 3 T Siemens Prisma Scanner (Siemens, Erlangen, Germany) at the Marseille MRI center (Centre IRM- INT@CERIMED, UMR7289 CNRS & AMU, http://irmf.int.univ-amu.fr/) using a 64-channel head coil. T1-weighted images were acquired using an MPRAGE sequence (voxel size = 1 × 1 × 1 mm^3^, data matrix = 256 × 256 × 192, TR/TI/TE = 2300/900/2.98 ms, flip angle = 9º). Fieldmap images were obtained using Dual echo Gradient-echo acquisition (TR = 677 ms, TE1/TE2 = 4.92/7.38 ms, FOV = 210 × 210 mm^2^, voxel size = 2.2 × 2.2 × 2.5 mm^3^). Functional images were collected using a gradient EPI sequence (TR = 1224 ms, TE = 30 ms, 54 slices with a thickness of 2.5 mm, FOV = 210 × 210 mm^2^, matrix = 84 × 84, flip angle = 66º, multiband factor = 3). Auditory hardware channel was composed of the Sensimetrics S14 MR-compatible insert earphones with a Yamaha P-2075 power amplifier.

Pre-processing was conducted by using fMRIPrep 20.0.6^62^. For more details, see fMRIPrep’s documentation (https://fmriprep.org/en/20.0.6/workflows.html). The T1-weighted image was corrected for intensity non-uniformity with N4BiasFieldCorrection in ANTs, and used as T1w-reference throughout the workflow. The T1w-reference was then skull-stripped. The brain-extracted T1w was used for segmentation of cerebrospinal fluid (CSF), white-matter (WM) and gray-matter (GM) using fast (FSL 5.0.9). Volume-based spatial normalization to the standard MNI space was performed through nonlinear registration with antsRegistration, using brain-extracted versions of both T1w reference and the T1w template (MNI152NLin2009cAsym). For functional images, the fieldmap distortion correction was performed based on a phase-difference map. The functional images were then co-registered to the T1w reference using flirt (FSL 5.0.9) with the boundary-based registration with nine degrees of freedom. Head-motion parameters were estimated before any spatiotemporal filtering using mcflirt (FSL 5.0.9). Fieldmap distortion correction, head-motion correction, BOLD-to-T1w co-registration, and spatial normalization were carried out in a single interpolation step by composing all the pertinent transformations. The pre-processed BOLD data were then used to calculate several confounding time series, including framewise displacement (FD), the mean signals within the white matter and the CSF, and a set of principal components of white matter and CSF that were extracted by the aCompCor method^63^.

### Network construction

In order to construct brain networks, a set of 264 regions of interest (ROIs) was taken from Power et al.^35^. Each ROI is a sphere with 5 mm radius and contains 81 voxels. The 264 spherical ROIs covers the entire cerebral cortex, subcortical areas and the cerebellum. This set of ROIs were intersected with the group-averaged gray matter mask to exclude areas that are outside the gray matter. One ROI at right thalamus (MNI x = 9, y = − 4, z = 6) was removed due to no overlap with the group-averaged gray matter, resulting in a set of 263 ROIs. The left-vOT identified at the group level in the visual localizer task (see Tasks and Stimuli and Supplementary Results 3) was further included (center MNI x = − 47, y = − 55, z = − 17) in the set of ROIs, while one ROI at left Fusiform gyrus (MNI x = − 47, y = − 51, z = − 21) was removed because it overlapped with the group left-vOT, thus resulting in a final set of 263 ROIs.

Pre-processed functional data were scaled to percent of signal change and modeled by using the Least Squares — Separate (LSS) method^64^ (3dLSS in AFNI) which ran a GLM for each trial and output trial-wise estimates (i.e., β coefficients) for the baseline and each speech processing condition. To estimate the edges of the networks in the baseline and speech processing conditions, we used a beta-series connectivity analysis^31^, which estimates functional connectivity between two regions by calculating the correlation of the activity (i.e., beta estimates) of the two regions across trials. Specifically, the six motion parameters, their temporal derivatives, and all their corresponding squared time series (i.e., 24 head motion regressors) were included in the LSS models to control for the impacts of head motion. In addition, the mean time-series and the first twelve principal components of white matter and of CSF were extracted by using the aCompCor method^63^ and used as nuisance regressors in the LSS models to reduce influence of physiological noise. The cosine-basis regressors estimated by fMRIPrep for high-pass filtering were also included in the LSS models as nuisance regressors. Motion contaminated volumes were identified by using framewise displacement (FD) and were censored along with the prior volume if their FD > 0.5 mm. On average, 2.2% of the volumes were censored. The trial-wise beta estimates were then used to calculate beta-series connectivity^31^. In line with the aim of the study, beta-series connectivity allows us to estimate functional connectivity between brain regions through characterizing the covariance between the brain regions' responses in different processing conditions^65^. In other words, it does not treat co-activation as a confounder but as part of connectivity and it still captures the connectivity between areas that have similar fluctuations of beta-series even when those areas are not significantly activated. Here we used beta-series connectivity to examine the left-vOT’s functional connectivity for each speech processing condition and to compare its connectivity profile between conditions.

For the baseline and each condition per participant, 80 beta estimates were obtained at each voxel with the LSS models; the series of beta estimates were then averaged over voxels within each ROI. Fisher-z-transformed Spearman correlation of averaged beta series between each pair of ROIs was then calculated, resulting in an undirected 263 × 263 correlation matrix. Each correlation matrix was thresholded into a binary matrix at a target density *d*% by keeping the *d*% of strongest edges as 1 and other edges as 0. The range of density between 15 and 22% (at intervals of 1%) was selected based on the largest connected component (LCC), which was calculated to ensure that the networks are largely connected since networks tend to be unstable and contain more fragments at lower densities, while they become more random as more noisy connections added at higher densities^66^. The lower bound 15% density is the sparsest density at which 90% of all the networks are fully-connected (i.e., the size of the LCC equals the size of the network), without any significant between-condition difference in the size of the LCC. The upper bound 22% density is the minimal density where all the networks are fully-connected. The results at the lower bound 15% were reported in the main text. The Supplementary Results 1 reported the results across the range of densities (15%-22%), with the false discovery rate (FDR) correction, to ensure that the results did not rely on a single density.

### Network analysis

Focusing on the left-vOT node, the graph theoretical analysis was carried out to characterize the topological organization of the brain network at the global scale and the nodal topology of the left-vOT at nodal scale (see Supplementary Table S1 for the interpretations of graph measures). The analysis was applied to the baseline and each condition per participant.

Firstly, to examine the global changes induced by speech processing compared to baseline, two global graph measures, i.e., *global efficiency* and *clustering coefficient*, were estimated to characterize the functional integration and segregation of the whole-brain network, respectively. *Global efficiency* is a measure of the capacity of the network for global information transfer. In other words, it measures information exchange between nodes by multiple parallel paths across the whole network. *Clustering coefficient* is a measure of the local efficiency of information transfer by parallel paths between the nearest neighbors of nodes^29^.

Secondly, three nodal measures, including *flow coefficient*, *betweenness centrality* and *participation coefficient* were estimated to characterize the functional role of the left-vOT node in the whole brain network during speech processing. *Flow coefficient* estimates the capacity of a node to transfer information between its neighbors. Nodes with large *flow coefficient* are identified as “local bridges” in the network^32^. *Betweenness centrality* estimates how often a node joins the shortest path between pairs of nodes in the network^33^. Nodes with large *betweenness centrality* are identified as “global bridges” in the network. *Participation coefficient* expresses the number of connections linked to a given node across different sub-networks. Node with high *participation coefficient*, that is a “connector”, plays a central role in coordinating the communication between different sub-networks^33,34^.

To estimate *participation coefficient*, sub-networks were first identified by using community detection to subdivide the whole brain network into several sub-networks through relatively maximizing intra-connections and minimizing inter-connections. Specifically, for each participant and each speech processing condition as well as the baseline, the Louvain algorithm^67^ and the consensus partitioning^68^ were applied on the whole brain network to determine the optimal partition, the corresponding *number of communities*, and *modularity Q*. The Louvain algorithm (γ = 1) was performed 1,000 times on the network to generate 1,000 initial optimal partitions that were used to estimate the agreement matrix, which was then submitted into the consensus partitioning (τ = 0.5, N_iter_ = 1,000) to converge to an optimal partition. The optimal partitions were then used for calculating *participation coefficient*. Additionally, the optimal partitions from different participants were further grouped and submitted into the consensus partitioning again to converge to a single representative partition for each condition. The representative partition of each speech processing condition as well as the baseline was used to identify all the sub-networks and the one to which the left-vOT node belonged. The anatomical locations of the sub-networks were visually inspected and labeled, by comparing them with the community structures defined by Power et al.^35^.

The network analysis was carried out by using the Brain Connectivity Toolbox^30^. To assess the significance of these graph measures, the repeated measures permutation test (Asymptotic General Independence Test) was adopted to compare multiple conditions, and the pairwise permutation test was used as a post-hoc test. The statistical tests were conducted by using R, the “coin” package (http://coin.r-forge.r-project.org/) and the “rcompanion” package (http://rcompanion.org/). In addition, to confirm the main results, the analyses described above were conducted again by using a symmetrical set of ROIs^69^ (see Supplementary Results 1).

## Supplementary Information

Supplementary Information.

## Data Availability

The datasets generated and/or analysed during the current study are not publicly available since the ethical approval for this study does not include permission to share data in a public data repository but are available from the corresponding author on reasonable request.
