# Supplementary material for: Graph theoretical analysis reveals the functional role of the left ventral occipito-temporal cortex in speech processing
Source: Sci Rep. 2022 Nov 21;12:20028. doi: 10.1038/s41598-022-24056-1 (PMC9681757; doi:10.1038/s41598-022-24056-1)
Supplement: Supplementary file 1 — Supplementary Information. [file 41598_2022_24056_MOESM1_ESM.pdf]

Title: Graph theoretical analysis reveals the functional role of the left ventral occipito-temporal cortex in speech processing

Shuai Wang[a,b], Samuel Planton[a,d], Valérie Chanoine[a,b], Julien Sein[c], Jean-Luc Anton[c], Bruno Nazarian[c], Anne-Sophie Dubarry[a], Christophe Pallier[d], Chotiga Pattamadilok[a]

[a] Aix Marseille Univ, CNRS, LPL, Aix-en-Provence, France

[b] Aix Marseille Univ, Institute of Language, Communication and the Brain, Aix-en-Provence, France

[c] Aix Marseille Univ, CNRS, Centre IRM-INT@CERIMED, Institut des Neurosciences de la Timone – UMR 7289, Marseille, France

[d] Cognitive Neuroimaging Unit, INSERM, CEA, CNRS, Université Paris-Saclay, NeuroSpin center, Gif/Yvette, France

Corresponding author
Chotiga Pattamadilok, PhD.
Laboratoire Parole et Langage
Centre National de la Recherche Scientifique (UMR 7309)
5, Av. Pasteur
13100 Aix-en-Provence, France
Email: chotiga.pattamadilok@univ-amu.fr
Tel : +33 601323435

Supplementary Information

Supplementary Table S1. Interpretations of graph theory terms.

Graph terminology	Topological scale	Interpretation
Global efficiency	global	Functional integration. Expresses information exchange between nodes by multiple parallel paths across the whole network.
Clustering coefficient	global	Functional segregation. Expresses information exchange by parallel paths between the nearest neighbors of nodes.
Modularity Q	sub-network	How well a network can be subdivided into non-overlapping sub-networks.
Community structure	sub-network	Sub-networks identified by community detection algorithm (partitions).
Flow coefficient	nodal	How well a node can transfer information between its neighbors. Hubs with large *flow coefficient* are identified as *local bridges* in the network.
Betweenness centrality	nodal	How often a node joins the shortest path between pairs of nodes in the network. Hubs with large *betweenness centrality* are identified as *global bridges* in the network.
Participation coefficient	nodal	How well a node can coordinate the communication between different sub-networks. Expresses the number of connections linked to a given node across sub-networks. Hubs with large *participation coefficient* are identified as *connectors* between sub-networks in the network.

Supplementary Table S2. A ROI within the primary visual cortex (V1) and a ROI in the left posterior STG (pSTG), the latter being involved in both spoken and written language processing[1-3], were selected from the set of 263 ROIs[4] as control regions to be compared with the left-vOT. Both V1 and pSTG showed lower ranks and significantly lower values for the three nodal measures of interest compared to the left-vOT (the p-value is shown in the brackets), except for *participation coefficient* in the CN+ condition, where the left-vOT itself did not act as a connector.

	Flow Coefficient				Betweenness				Participation			
	PN-	PN+	CN-	CN+	PN-	PN+	CN-	CN+	PN-	PN+	CN-	CN+
left-vOT	3	13	5	12	7	1	35	5	1	1	3	40
pSTG	84 (0.013)	77 (0.0058)	109 (5.2e-4)	141 (0.0056)	215 (3.7e-4)	198 (4.5e-4)	232 (0.0013)	226 (1.4e-4)	50 (0.018)	37 (0.017)	54 (0.012)	132 (0.11)
V1	225 (5.6e-4)	205 (7.1e-4)	251 (6.3e-4)	191 (0.011)	234 (1.2e-4)	211 (3e-4)	235 (8.8e-4)	222 (1.6e-4)	160 (0.0014)	156 (0.0017)	212 (0.0011)	100 (0.24)

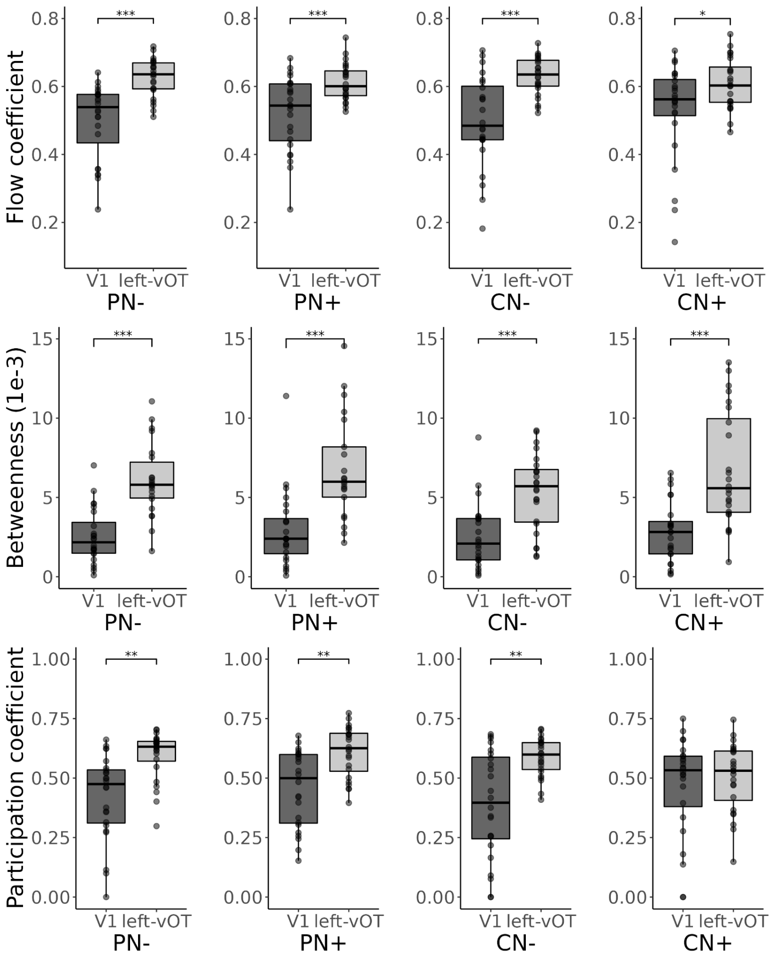

Supplementary Figure S1. The ROI of V1 (primary visual cortex; MNI x = -8, y = -81, z = 7) showed significantly lower values in *flow coefficient*, *betweenness centrality* and *participation coefficient* than the left-vOT for each condition, except for *participation coefficient* in the CN+ condition, where the left-vOT did not act as a connector. ***: $p < 0.001$; **: $p < 0.01$; *: $p < 0.05$

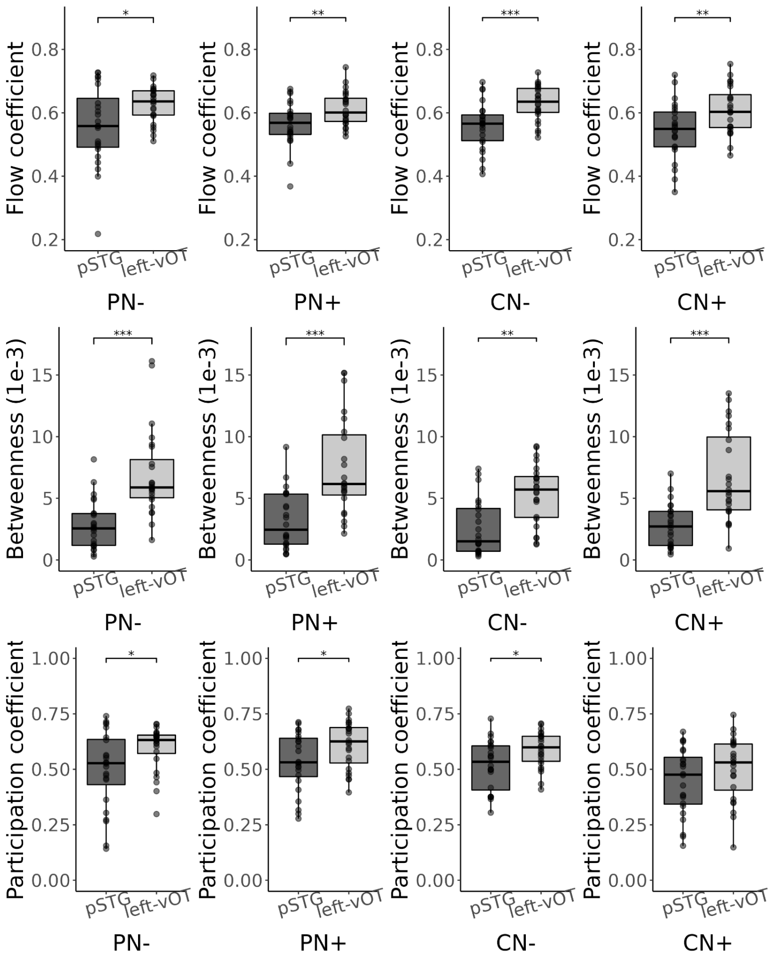

Supplementary Figure S2. The ROI of pSTG (MNI x = -55, y = -40, z = 14) showed significantly lower values in *flow coefficient*, *betweenness centrality* and *participation coefficient* than the left-vOT for each condition, except for *participation coefficient* in the CN+ condition, where the left-vOT did not act as a connector. ***: $p < 0.001$; **: $p < 0.01$; *: $p < 0.05$

Supplementary Results 1

To ascertain that the results reported in the main document, where the analyses was conducted on the ROIs from Power et al.[4], were not restricted to a specific set of ROIs or to a single density (15%), we conducted additional analyses: 1) across the range of densities 15%-22% with the FDR correction using Power et al.'s ROI[4] and 2) using the symmetrical ROIs set[5] across the range of densities 14%-18% with the FDR correction. As respectively shown in **Supplementary Tables S3** and **S4**, the new analyses confirmed the patterns of functional connectivity reported in the main document. Moreover, we also applied

community detection on the network density ranging from 2% to 10% with step 1% to confirm the stability of the modularity measure[4]. The result showed no differences in modularity Q across the baseline and four speech processing conditions (**Supplementary Table S5**; all ps > 0.39), indicating a stable community structure across the range of sparser network density.

Network construction of the symmetrical ROIs set. A symmetrical set of 402 ROIs were taken from Di et al.[5] to confirm the global and nodal results reported in the main text while controlling the spatial bias in lateralization. The same procedure as described in the main text (see Network Construction) was performed on this set of ROIs. Specifically, the 402 ROIs with a radius of 5 mm were intersected with the group-averaged gray matter mask to exclude the ROIs that are outside the gray matter, resulting in 392 ROIs. The 10 ROIs that were removed were mainly subcortical nuclei located outside the gray matter mask. Additionally, the left-vOT identified in the visual localizer task and its homogeneous region in the right hemisphere were included into the symmetrical ROIs set. Finally, three ROIs in the left Fusiform gyrus and three ROIs in right Fusiform gyrus were further removed due to the overlap with functionally localized left-vOT and its homogeneous region, respectively. Altogether, this resulted in 388 ROIs in the symmetrical set (see **Supplementary Fig. S3** for the spatial distribution of this set of ROIs). The beta-series connectivity and Fisher-z transformation were then applied on the symmetrical ROIs set to construct a brain network for each condition and each participant. The density range here was identified between 14% and 18% at steps of 1%. The lower bound, 14% density, is the sparsest density at which 90% of all the networks are fully-connected, without any significant between-condition difference in the size of the LCC. The upper bound, 18% density, is the minimal density where all the networks are fully-connected networks.

Global network measures. As a demonstration, the results obtained using the symmetrical ROIs set at the sparsest density 14% are shown here to validate the results reported in the main document. At the global scale, the *post-hoc* tests confirmed that the *global efficiency* in the PN- ($p < 0.040$), CN- ($p < 0.00066$) and CN+ ($p < 0.0036$) conditions was significantly higher than in the baseline, while the difference between the PN+ and the baseline was marginally significant ($p < 0.053$). In terms of *clustering coefficient*, the *post-hoc* tests confirmed that the CN- ($p < 0.0018$) and CN+ ($p < 0.0026$) conditions are significantly lower than the baseline, while the difference between the baseline and the perception conditions are

not significant (all *p*s > 0.083). Overall, the results confirmed that the speech processing led to higher *global efficiency* and lower *clustering coefficient* compared to the baseline.

Nodal measures of the left-vOT. At the nodal scale, hubs are identified as the top 5% nodes among the 388 nodes (i.e., above 19th out of 388). **Supplementary Fig. S4A** showed the rank of vOT in terms of *betweenness centrality* and *participation coefficient*. Among the 388 nodes, the left-vOT node was ranked 4th (PN-), 4th (PN+), 71st (CN-) and 14th (CN+) in order of *betweenness centrality*, and ranked 1st (PN-), 3rd (PN+), 11th (CN-) and 45th (CN+) in order of *participation coefficient*. The *post-hoc* tests confirmed that the *participation coefficient* in the CN+ condition was significantly lower than in the PN- ($p < 0.0099$) and the PN+ ($p < 0.026$) conditions, but not the CN- condition (**Supplementary Fig. S4B**). However, in terms of *flow coefficient*, the left-vOT was not identified as a hub since its ranks were below 35th in all conditions. It is worth noting that this set of ROIs are data driven and strictly symmetrical[5], which is not the case for the set of ROIs from Power et al.[4]). The bilaterally homologous regions tend to be strongly connected[6,7], therefore, this set of ROIs can induce numerous one-step connections between mirror regions and result in reorganized local bridges in the network. As shown in **Supplementary Fig. S4C**, the correlation analysis showed that *participation coefficient* and reaction time was negatively correlated in the CN+ condition (Pearson's r = -0.51, $p < 0.011$), while the correlations were not significant in other conditions (all *p*s > 0.32).

Supplementary Table S3. Between-conditions significance in global and nodal metrics of brain networks based on the 263 ROIs[4]. The results were corrected with FDR across the range of densities (15%-22%). FDR corrected ps < 0.05 are highlighted in bold. Comparisons conducted on global metrics include the baseline and four speech processing conditions, while comparisons conducted on nodal metrics include the four speech processing conditions.

	Density							
	**15%**	**16%**	**17%**	**18%**	**19%**	**20%**	**21%**	**22%**
Global efficiency	**0.005**	**0.005**	**0.005**	**0.005**	**0.005**	**0.005**	**0.005**	**0.005**
Clustering coefficient	**0.009**	**0.009**	**0.009**	**0.009**	**0.009**	**0.009**	**0.009**	**0.009**
Number of communities	0.391	0.432	0.432	0.432	0.432	0.432	0.391	0.432
Modularity Q	0.426	0.426	0.426	0.426	0.426	0.426	0.426	0.426
Flow coefficient	0.670	0.670	0.670	0.670	0.670	0.670	0.670	0.670
Betweenness centrality	0.072	0.072	0.072	0.072	0.072	0.072	0.072	0.072
Participation coefficient	**0.002**	**0.002**	**0.002**	**0.002**	**0.004**	**0.028**	**0.028**	0.074

Supplementary Table S4. Between-conditions significance in global and nodal metrics of brain networks based on the 388 ROIs[5]. The results were corrected with FDR across the range of densities (14%-18%). FDR corrected ps < 0.05 are highlighted in bold. Comparisons conducted on global metrics include the baseline and four speech processing conditions, while comparisons conducted on nodal metrics include the four speech processing conditions.

	Density				
	**14%**	**15%**	**16%**	**17%**	**18%**
Global efficiency	**0.003**	**0.003**	**0.003**	**0.003**	**0.003**
Clustering coefficient	**0.003**	**0.003**	**0.003**	**0.003**	**0.003**
Number of communities	0.105	0.105	0.105	0.105	0.148
Modularity Q	0.795	0.795	0.795	0.795	0.795
Flow coefficient	0.577	0.416	0.416	0.416	0.416
Betweenness centrality	0.058	0.058	0.058	0.062	0.058
Participation coefficient	**0.050**	**0.046**	**0.046**	**0.046**	**0.050**

Supplementary Table S5. No significant differences across the baseline and the four speech processing conditions in modularity Q of brain networks based on the 263 ROIs[4], in the range of density 2% - 10%.

	Density								
	**2%**	**3%**	**4%**	**5%**	**6%**	**7%**	**8%**	**9%**	**10%**
Modularity Q	0.392	0.427	0.427	0.427	0.427	0.393	0.502	0.536	0.644

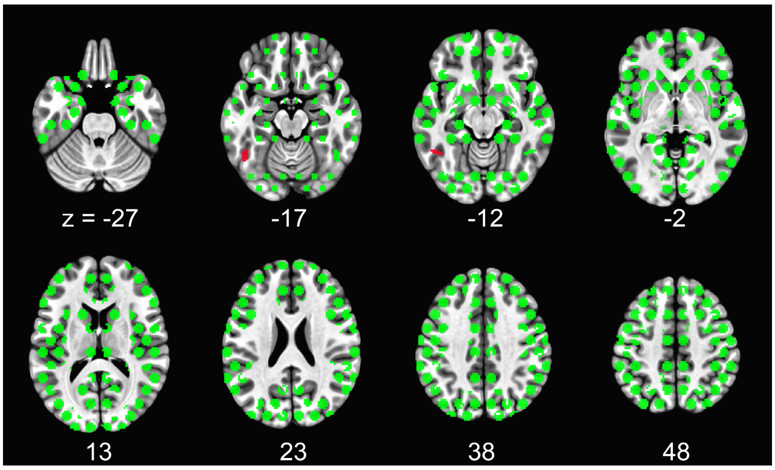

Supplementary Figure S3. The spatial distribution of the set of 388 ROIs originally taken from Di et al.[5]. *Notes:* Each ROI is a sphere with a radius of 5mm (i.e., 81 voxels) and is intersected with the group-averaged gray matter mask to exclude areas that are outside the gray matter. The group left-vOT with 81 voxels (colored in red) is centered at MNI x = -47, y = -55, z = -17.

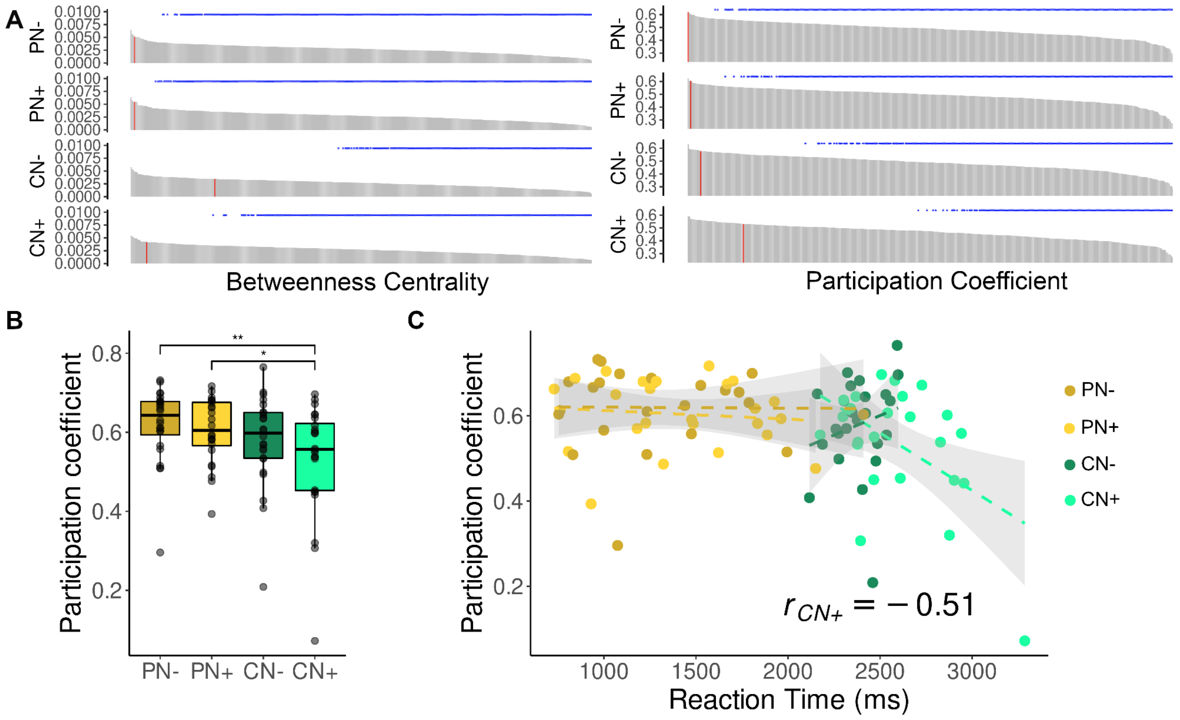

Supplementary Figure S4. A. Among all the 388 nodes, the left-vOT node showed high *betweenness centrality* in the PN-. PN+ and CN+ conditions, and high *participation coefficient* in the PN-, PN+ and CN+ conditions. The blue asterisks indicate the nodes whose values were significantly lower than the left-vOT's (paired permutation test, $p < 0.05$ unc.). **B.** The *participation coefficient* of the left-vOT node was significantly lower in the CN+ condition than in the PN- and PN+ conditions, but not the CN- condition. **C.** The *participation coefficient* and reaction time was negatively correlated in the CN+ condition (light green dots and dashed line; Pearson's r = -0.51, $p < 0.011$), while the correlations are not significant in other conditions (all *p*s > 0.32).

Supplementary Results 2

The analysis of community detection identified four sub-networks for both baseline and speech processing conditions. As mentioned in the Results section of the main text, the community structures were similar across conditions. We labeled these four sub-networks as visual network, fronto-parietal network, default mode network, and sensorimotor-auditory network based on the original community structure and labels defined by Power et al.[4]. As illustrated in **Supplementary Fig. S5**, the cyan and dark blue sub-networks highly matched the "visual network" and "default mode network" reported in Power et al.[4], respectively. The

green sub-network largely overlapped with the fronto-parietal task control, ventral and dorsal attention, and salience networks in Power et al.[4] and was labeled “fronto-parietal network” in our study. Finally, the magenta sub-network contained two somatosensory-motor networks (month and hand) and the auditory network reported in Power et al.[4]. Although it also overlapped with several subcortical networks (i.e., cingulo-opercular task control, subcortical, cerebellar), we labeled this sub-network as “Sensorimotor-auditory Network”.

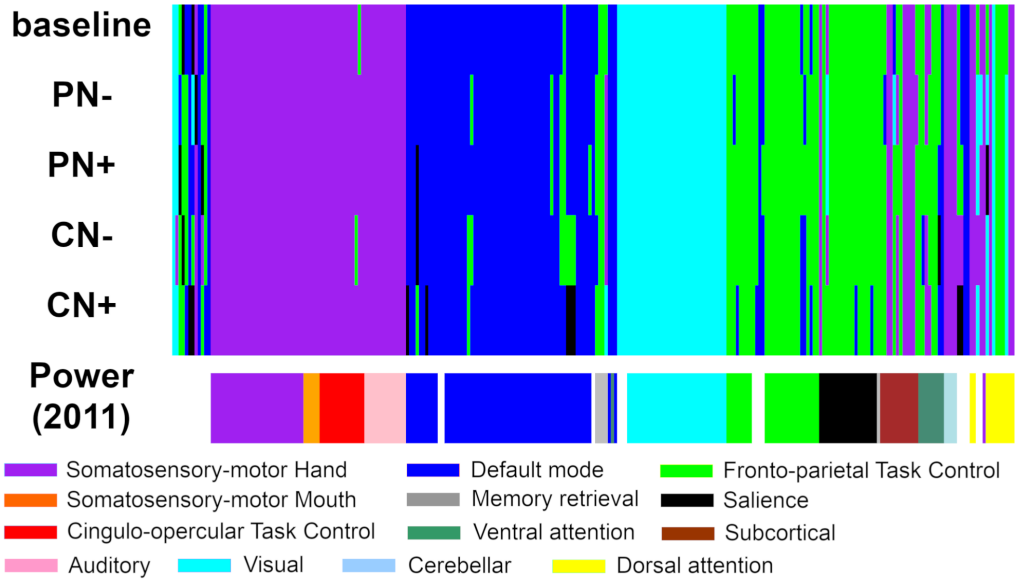

Supplementary Figure S5. Community structures (partitions) and labeling. The top five rows show the community structures identified for baseline and speech processing conditions, which mainly contain four sub-networks that are colored cyan (visual network), green (fronto-parietal network), dark blue (default mode network) and magenta (sensorimotor-auditory network). The bottom row shows the community structure defined by Power et al.[4] through meta-analysis, where white bars present ROIs that cannot be clearly assigned into a sub-network. The legend shows the labels originally defined by Power et al.[4].

Supplementary Results 3

Group left-vOT defined by the visual localizer task. The pre-processed functional data obtained in the visual localizer task with a block-design were fitted into a general linear model (GLM) using AFNI. The same set of nuisance regressors as the spoken sentence

processing tasks were included, that are 1) 24 head motion regressors: the six motion parameters, their temporal derivatives, and all their corresponding squared time series and, 2) 26 physiological regressors: the mean time-series and the first twelve principal components of white matter and of CSF which were extracted by using the aCompCor method, as well as the cosine-basis regressors estimated by fMRIPrep. Motion contaminated volumes were identified by using framewise displacement (FD) and were censored along with the prior volume if their FD > 0.5mm. On average, 1.8% of the volumes were censored for the visual localizer task. The group level analysis was then performed to compute the contrast *words - consonant strings* by using pairwise t-test in AFNI (3dttest++). To combine the group left-vOT with the set of 263 spherical ROIs[4] that are described in the main text, the group left-vOT with the same volume of 81 voxels as a spherical ROI was extracted. To this aim, the group activation map of the contrast *words - consonant strings* was thresholded to extract the strongest cluster with a volume of 81 voxels by adjusting the significance threshold ($T > 5.2536$, $p < 2.5e-5$). The spatial distribution of the final set of ROIs is shown in **Supplementary Fig. S6**.

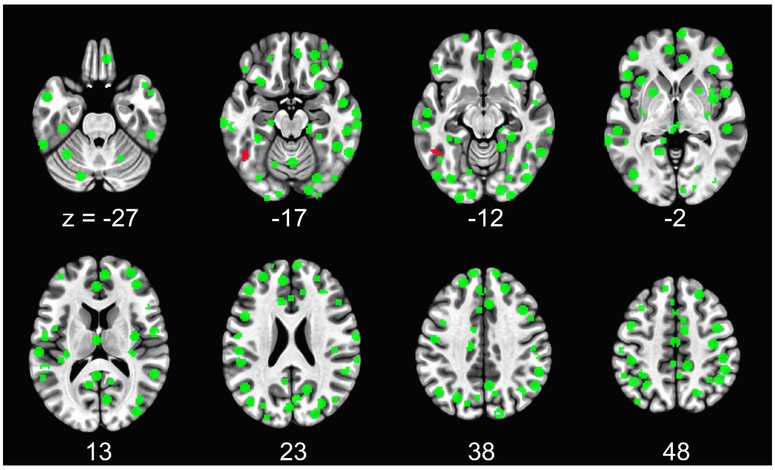

Supplementary Figure S6. The spatial distribution of the set of 263 ROIs originally taken from Power et al.[4]. *Notes:* Each ROI is a sphere with a radius of 5mm (i.e., 81 voxels) and is intersected with the group-averaged gray matter mask to exclude areas that are outside the gray matter. The group left-vOT with 81 voxels (colored in red) is centered at MNI x = -47, y = -55, z = -17.

Supplementary References

1. Price, C. J. A review and synthesis of the first 20 years of PET and fMRI studies of heard speech, spoken language and reading. *Neuroimage* **62**, 816–47 (2012).
2. Monzalvo, K. & Dehaene-Lambertz, G. How reading acquisition changes children's spoken language network. *Brain Lang.* **127**, 356–365 (2013).
3. Rueckl, J. G. *et al.* Universal brain signature of proficient reading: Evidence from four contrasting languages. *Proc. Natl. Acad. Sci. U. S. A.* **112**, 201509321 (2015).
4. Power JD, Cohen AL, Nelson SM, Wig GS, Barnes KA, Church JA, Vogel AC, Laumann TO, Miezin FM, Schlaggar BL, Petersen SE. 2011. Functional network organization of the human brain. Neuron. 72:665–678.
5. Di X, Kim EH, Chen P, Biswal BB. 2014. Lateralized resting-state functional connectivity in the task-positive and task-negative networks. Brain Connect. 4:641–648.
6. Salvador R, Suckling J, Schwarzbauer C, Bullmore E. 2005. Undirected graphs of frequency-dependent functional connectivity in whole brain networks. Philos Trans R Soc B. 360:937–946.
7. Mancuso L, Costa T, Nani A, Manuello J, Liloia D, Gelmini G, Panero M, Duca S, Cauda F. 2019. The homotopic connectivity of the functional brain: a meta-analytic approach. Sci Rep. 9:1–19.
